# Directed Differentiation of Adult Liver Derived Mesenchymal Like Stem Cells into Functional Hepatocytes

**DOI:** 10.1038/s41598-018-20304-5

**Published:** 2018-02-12

**Authors:** Xiaobei Luo, Kapish Gupta, Abhishek Ananthanarayanan, Zenan Wang, Lei Xia, Aimin Li, Rashidah Binte Sakban, Side Liu, Hanry Yu

**Affiliations:** 1 Department of Gastroenterology, Nanfang hospital, Southern Medical University, Guangzhou, China; 2 Mechanobiology Institute, National University of, Singapore, Singapore; 3 Invitrocue Pte Ltd, Singapore, Singapore; 4 Institute of Bioengineering and Nanotechnology, Agency for Science, Technology and Research (A*STAR), Singapore, Singapore; 5 NUS Graduate School for Integrative Sciences and Engineering, Centre for Life Sciences (CeLS), Singapore, Singapore; 6 Department of Gastroenterology, Beijing Chao-Yang Hospital, Capital Medical University, Beijing, China; 7 Department of Physiology, Yong Loo Lin School of Medicine, National University of Singapore, Singapore, Singapore; 8 BioSyM, Singapore-MIT Alliance for Research and Technology, Singapore, Singapore

## Abstract

Shortage of functional hepatocytes hampers drug safety testing and therapeutic applications because mature hepatocytes cannot be expanded and maintain functions *in vitro*. Recent studies have reported that liver progenitor cells can originate from mature hepatocytes *in vivo*. Derivation of proliferating progenitor cells from mature hepatocytes, and re-differentiation into functional hepatocytes *in vitro* has not been successful. Here we report the derivation of novel mesenchymal-like stem cells (arHMSCs) from adult rat hepatocytes. Immunofluorescence and flow cytometry characterization of arHMSCs found expression of mesenchymal markers CD29, CD44, CD90, vimentin and alpha smooth muscle actin. These arHMSCs proliferated *in vitro* for 4 passages yielding 10^4^ fold increase in cell number in 28 days, and differentiated into hepatocyte-like cells (arHMSC-H). The arHMSC-H expressed significantly higher level of hepatocyte-specific markers (200 fold for albumin and 6 fold for Cyp450 enzymes) than arHMSCs. The arHMSC-H also demonstrated dose response curves similar to primary hepatocytes for 3 of the 6 paradigm hepatotoxicants tested, demonstrating utility in drug safety testing applications.

## Introduction

Liver is an important organ for maintaining homeostasis and performs a plethora of functions such as protein synthesis and metabolism, conversion of ammonia to urea, glucose, fatty acid and drug metabolism. Hepatocytes, the parenchymal cells of the liver, are widely used in bio-artificial liver devices and for safety testing of xenobiotics *in vitro*^1–3^. However, there is a shortage of functional hepatocytes for various drug-testing applications and large quantities of hepatocytes are unavailable from a single donor that is crucial for toxicity studies and infectious disease applications. In recent years embryonic stem cells (ESCs) and induced pluripotent stem cells (iPSCs) have been shown to differentiate into multiple lineages including hepatocytes like cells^4^. However, proliferation and maintenance of these cultures in their pluripotent state is expensive and difficult to scale up.

Liver progenitor cells have also garnered great interest from both the biomedical and pharmaceutical industries due to their potential for providing an unlimited cell source of cells for various applications^5^. The oval cells are a well-described type of liver progenitor cells but are difficult to isolate and maintain in culture^6^. Previous studies have demonstrated that liver-derived progenitor cells isolated from diseased liver of rodents such as fibrotic liver and hepatocellular carcinoma tissues have the capacity to differentiate into hepatocytes *in vitro*^7,8^. Recent studies have shown that, under some conditions mature hepatocytes can differentiate into oval cells *in vivo*, which in turn can proliferate and differentiate to hepatocytes *in vivo*. However, such potential of hepatocytes to give rise to progenitor cells capable of proliferating and differentiating into hepatocytes has not been achieved *in vitro* and applied for drug-testing applications^9–12^. In this study we have derived adult rat hepatocyte-derived mesenchymal-like stem cells (arHMSCs) from rat hepatocytes, and re-differentiated them into hepatocyte-like cells and characterized them for drug-testing applications^13,14^.

arHMSCs in our study were positive for mesenchymal markers such as alpha-smooth muscle actin (SMA), Vimentin, CD44, CD29 and CD90. arHMSCs were also vastly different in morphology compared to the liver-derived progenitor cells (LDPCs) derived by Sahin *et al*.^14^ and did not express CD45. arHMSCs were derived directly from hepatocytes but not from the clonal selection and LDPC repopulation as described by Sahin and Chen *et al*.^13,14^.

We differentiated arHMSCs using chemically defined media and quantified the expression and metabolic activity of the arHMSCs by studying the expression of cytochrome P450 markers and sensitivity of arHMSCs to 6 paradigm hepatotoxicants. Over fourteen days in culture arHMSCs differentiated to an epithelial lineage and expressed liver-specific phenotypes such as ureagenesis, albumin secretion, expression of hepatic nuclear factors and cytochrome P450 enzymes. They also exhibited dose-dependent hepatotoxicity similar to that seen in mature hepatocytes for half of the tested hepatotoxicants. This was contrary to the results obtained by Maerckx *et al*. where they failed to differentiate the liver progenitor cells to mature hepatocytes^15^ though the progenitor cells were of similar morphology to our study.

arHMSC-H in our study demonstrated hepatocyte-like liver-specific functions and also exhibited dose-dependent toxicity to selected paradigm hepatotoxicants, with dose response curves similar to that observed in mature hepatocytes. We have developed a simple method to generate functional hepatocyte-like cells *in vitro* using progenitor cells derived from adult hepatocytes. By bypassing embryonic stem cell reprogramming and avoiding the use of viral vectors in induced pluripotent stem cells, our approach can be adapted to generate patient-specific progenitors for individualized toxicity screens.

## Results

### Derivation of arHMSCs from primary rat hepatocytes

**Figure 1:**
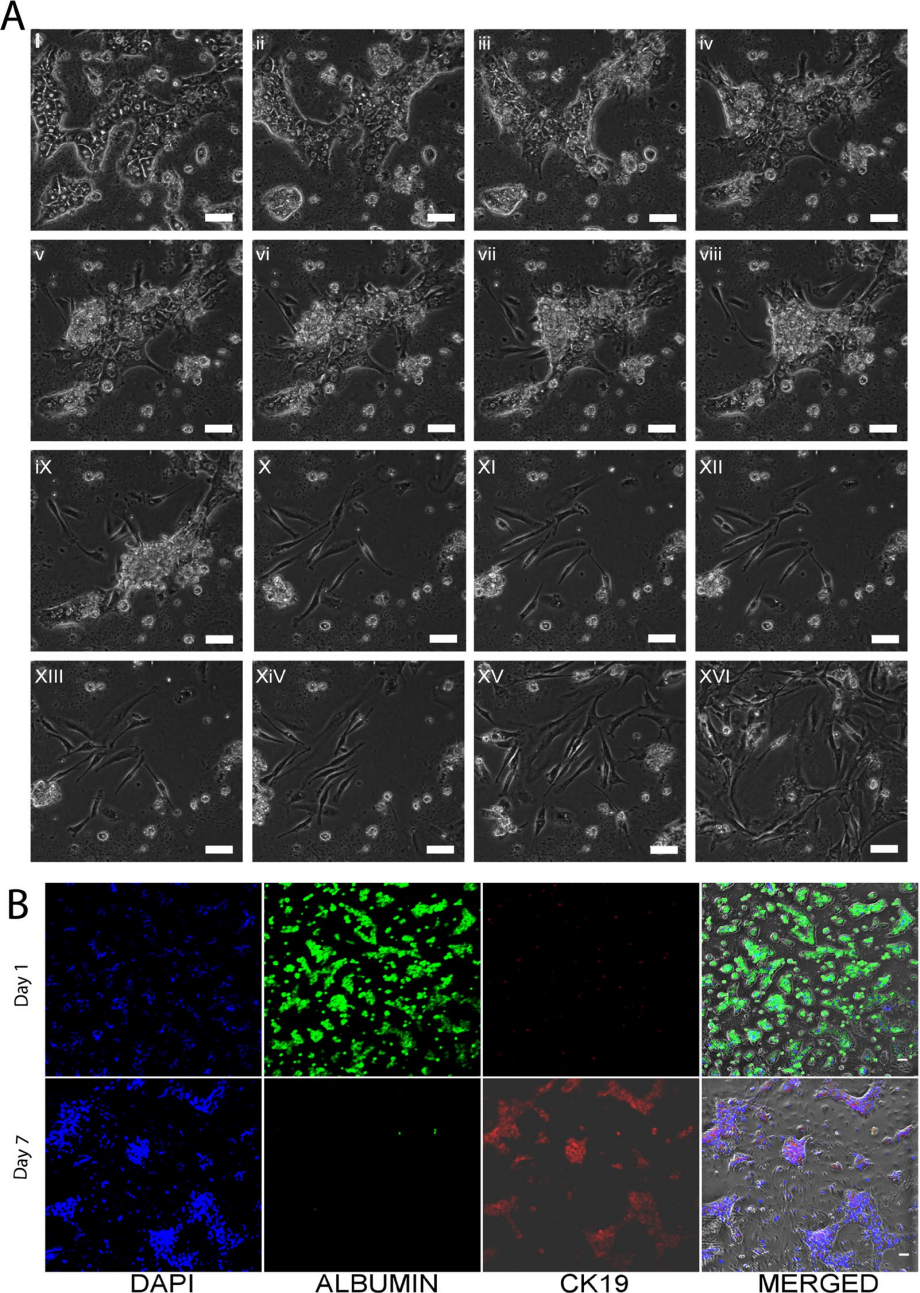
Time lapse imaging of transformation of primary rat hepatocytes to arHMSCs. A (i–iv) Phase contrast image of the dedifferentiating hepatocytes on day 3 & day 4 of culture. The hepatocytes lose their cuboidal morphology and the hepatocyte islands start to shrink and round up with a gradual loss of bile-canaliculi like structures. (**A**) (v–viii) Phase contrast image of dedifferentiating hepatocytes on day 5 of culture over a one-hour (1) time scale. (Scale bar = 100 μM). This step involves the further shrinkage and aggregation of the hepatocyte islands and migration and merging of two separate hepatocyte islands. It is also characterized by the appearance of cells with mesenchymal like morphology from the regions where hepatocyte islands had clumped and formed spheroid like structures. A (ix-xii) Phase contrast images of complete transformation of hepatocytes to ALMLCs in culture by day 6. This step is characterized by the complete disappearance of hepatocyte aggregates in culture (Scale bar = 100 μM). A(xiii-xvi) Phase contrast image of proliferation of ALMLCs in culture on day 7. This step is characterized by the proliferation of ALMLCs in culture. The ALMLCs rapidly proliferate in culture and give rise to increasing number of fibroblast like cells. (**B**) Staining of primary hepatocytes and the dedifferentiated hepatocytes on Day 1 and Day 7 for mature hepatocyte marker Albumin (Green), early fetal hepatocyte marker CK 19 (Red) and DAPI (blue).

We studied the morphological changes in the primary rat hepatocytes cultured for seven days in culture using continuous live cell imaging. We did not observe morphological changes in the primary rat hepatocytes on day 1 and 2 of culture. Live cell imaging was performed from day 3 onwards. Mature hepatocytes started to undergo distinct morphological changes from day 3 in culture (Fig. 1(i–iv)). Some hepatocytes aggregated to form clusters, parts of which detached from the culture plate. From day 5 onwards, mesenchymal-like elongated spindle cells, migrated away from the attached, aggregated hepatocyte clusters (Fig. 1(v–viii)). At the same time, more hepatocytes aggregated to form spheroids and some detached from the plate. By day 6, there were only a few hepatocyte clusters left and the number of spindle shaped cells increased (Fig. 1(ix–xii)). Upon longer culture, the spindle shaped cells further increased in number (Fig. 1(xiii–xvi)). These spindle shaped cells were denoted as arHMSCs. We also stained the primary hepatocytes on day 1 of culture and on day 7 post isolation in culture. The cells were positive for albumin, a mature hepatocyte specific marker on day 1 of culture and negative for CK 19, which is an early fetal hepatocyte marker. Upon de-differentiation, the cells were negative for the mature hepatocyte marker albumin but were positive for the fetal hepatocyte marker CK 19 on day 7 in culture (Fig. 1B).

**Figure 2:**
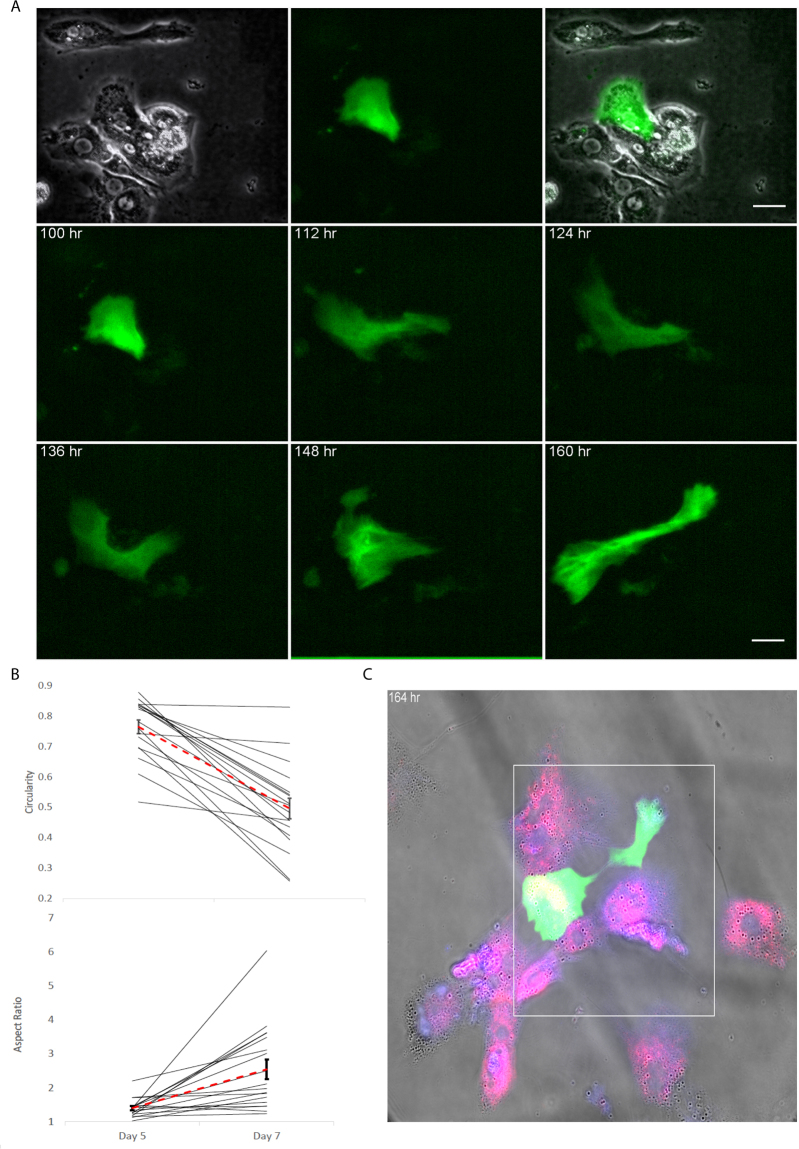
Single cell lineage tracking of transformation of hepatocytes to arHMSCs. (**A**) Tracking the dedifferentiation of a single GFP labeled hepatocyte into arHMSCs over 7 days in culture. The cells lose their rounded hepatocyte-like morphology over time and become elongated mesenchymal like cells over 7 days in culture. (**B**) Measure of aspect ratio and circularity of the cells from Day 1 to Day 7 in culture upon dedifferentiation. The cells demonstrated decreasing circularity and increasing aspect ratio over time when undergoing dedifferentiation. (**C**) Staining of arHMSCs for CK19 (blue) and N Cadherin (red) on day 7 of culture. The cells were positive for both CK 19 and N-Cadherin, which are early fetal hepatocyte and mesenchymal markers.

One of the major concern was to delineate the source of these arHMSCs. Since the cells we used were isolated from rat liver with ~90 percent hepatocytes and used without any further purification, there was a fair chance that arHMSCs may emerge from cells other than hepatocytes. To circumvent this problem we used bile canaliculi as a morphological marker of hepatocytes. Hepatocytes can form distinct bile canaliculi, which in phase contrast microscopy appear as bright tubes between adjacent cobblestone-shaped cells (hepatocytes). We confirm the bright tubes as bile canaliculi by incubating with cholyl-lysyl-fluorescein (CLF), a bile salt analogue, which is excreted into the bile canaliculi from the surrounding hepatocytes (Supporting Fig. 1)^16^ when the hepatocytes are polarized. Thus, hepatocytes can be easily identified in culture by their ability to form bile canaliculi. To confirm that the arHMSCs originated from the mature primary hepatocytes in culture, we transfected the isolated cells with green fluorescent protein (GFP) – Ftractin (F-actin)^17^ construct and performed time-lapse imaging of individual GFP-Ftractin labeled hepatocyte (i.e. cells forming bile canaliculi) over 7 days. We observed that the hepatocytes maintained cobblestone morphology until day 4 of culture (Fig. 2A). From day 5 onwards, the hepatocytes started losing their circularity and became elongated to resemble mesenchymal-like cells (Supporting Fig. 2). By day 7 in culture the entire population in the well had elongated cells reminiscent of mesenchymal cells (Fig. 2A). This is also evident from changes the circularity and the aspect ratio of the cells from day 5 to day 7 where we observe a decrease in circularity and increase in aspect ratio of the cells upon dedifferentiation (Fig. 2B). Both, decrease in circularity and increase in aspect ratio are physical marker of epithelial to mesenchymal transition (EMT) process. Next, we stained the cells that we tracked for EMT, with progenitor markers. We observed that on day 7 of culture the tracked hepatocyte that have undergone changes in aspect ratio and circularity, and expressed N-Cadherin and CK 19, which are mesenchymal and fetal hepatocyte markers (Fig. 2C). These findings demonstrate that arHMSCs are derived from mature primary hepatocytes and exhibit characteristics of mesenchymal-like cells.

### Characterization of arHMSCs

**Figure 3:**
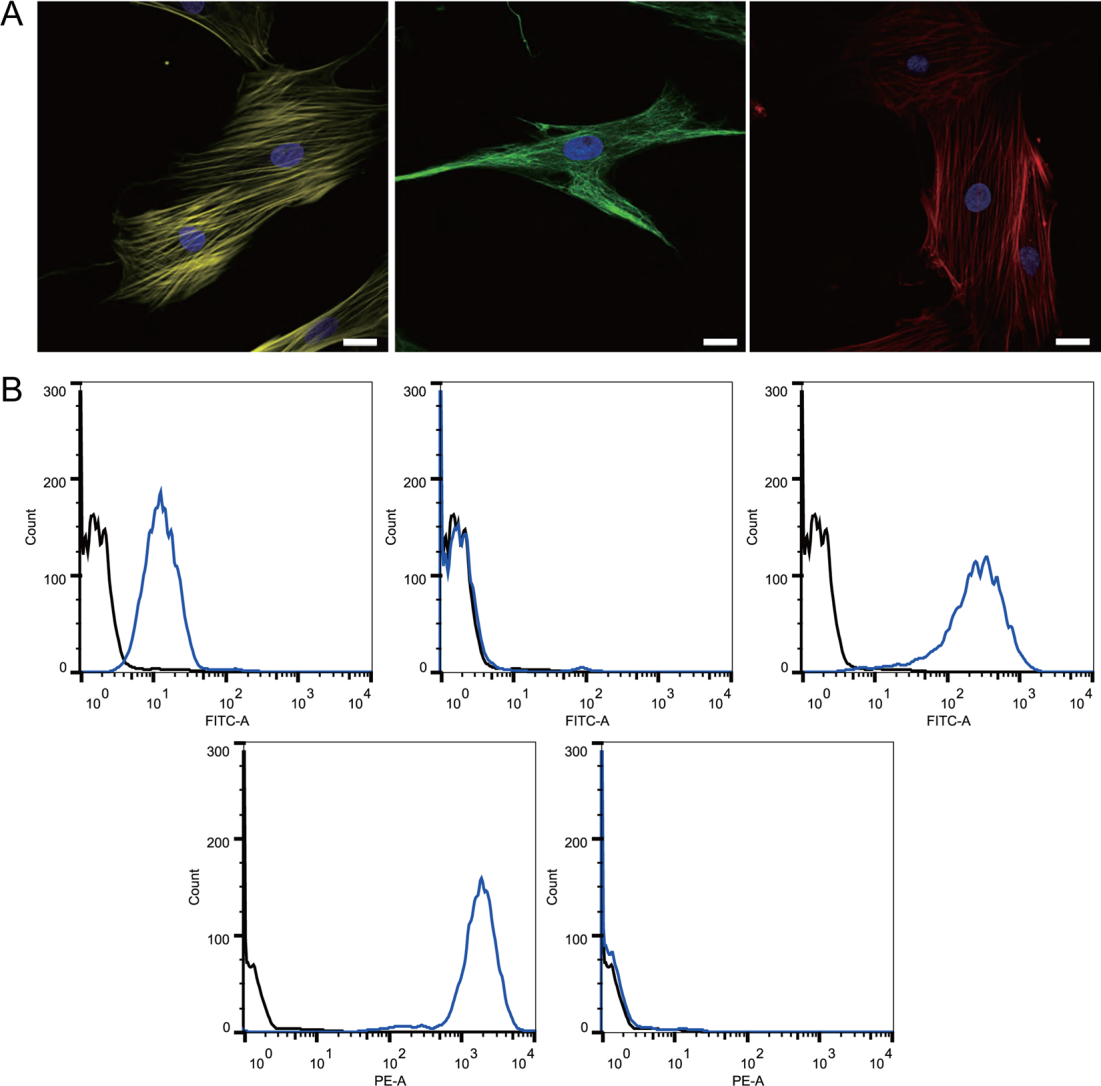
Characterization of arHMSCs. (**A**) Immunofluorescence staining of arHMSCs. arHMSCs were stained with vimentin (yellow), actin (green), α-SMA (Red) and DAPI (blue) fluorescence. The arHMSCs were positive for vimentin, actin and α-SMA, which are mesenchymal markers (Scale bar = 20 μM). (**B**) Flow cytometry analysis of arHMSCs. (Negative control: black; stained arHMSCs: Blue) The top left, middle and right graph represent CD44 CD45 and CD90 respectively. Bottom left and right graph represent CD29 and ASGPR. arHMSCs expressed mesenchymal markers CD29, CD44 and CD90. The hepatocyte specific marker ASGPR and hematopoietic marker CD45 were almost undetectable.

We examined the expression of commonly used mesenchymal markers using flow cytometry. The arHMSCs at passage 3 indicated that the cells expressed CD29, CD44 and CD90 with a positive population of 98.90% ± 1.25%, 21.36% ± 6.97% and 89.10% ± 6.67%, respectively (Fig. 3B). Additionally, the hepatocyte-specific markers ASGPR and hematopoietic marker CD45 were almost undetectable, with a positive population percentage of 2.21 ± 0.08% and 4.95 ± 5.16% respectively. arHMSCs did not express Albumin, a protein that is secreted by adult hepatocytes. To further characterize arHMSCs, we stained arHMSCs for α-SMA, actin and vimentin. arHMSCs exhibited cytoplasmic distribution of α-SMA similar to that found in mesenchymal cells. Additionally, extensive stress fibers were observed in arHMSCs as demonstrated by phalloidin staining (Fig. 3A), indicating strong cell substrate adhesion and functional dedifferentiation of hepatocytes^18^. arHMSCs also expressed vimentin, another mesenchymal marker^19^ (Fig. 3A). These results indicate that arHMSCs are mesenchymal-like cells.

### *In vitro* differentiation from arHMSCs to arHMSC-Hs

**Figure 4:**
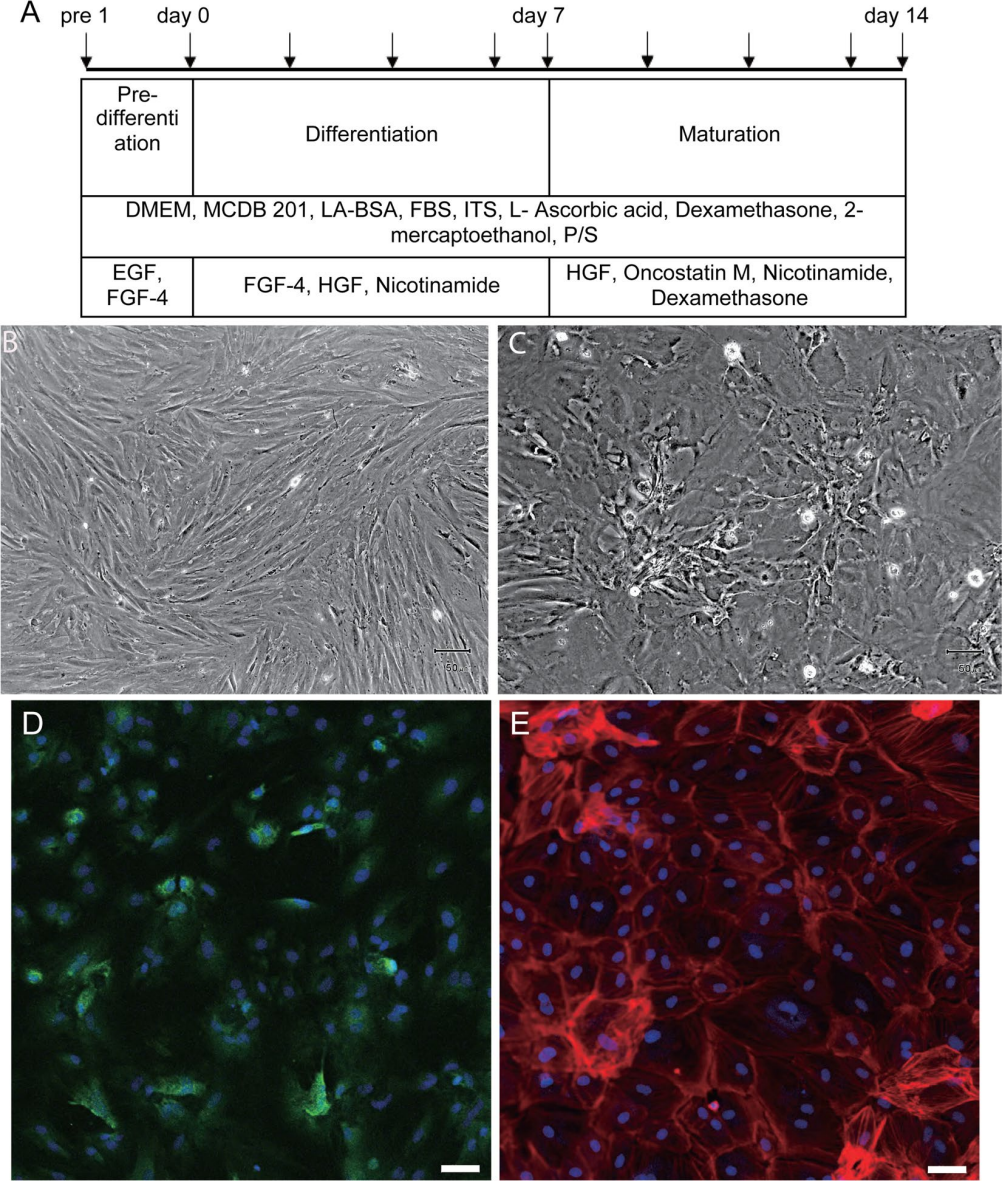
*In vitro* differentiation of arHMSCs. (**A**) Modified protocol of directed hepatogenic differentiation of arHMSCs. (**B**) Phase contrast image of undifferentiated arHMSCs, which exhibit spindle shaped cell morphology similar to that of MSCs (Scale bar = 100 μM). (**C**) Phase contrast image of differentiated arHMSCs after 14 days of *in vitro* differentiation. arHMSC-Hs adopted a polygonal morphology similar to that observed in epithelial cells (Scale bar = 100 μM). (**D**) Immunofluorescence of the arHMSC-Hs for albumin, a specific marker for hepatocytes, confirmed the hepatic differentiation of arHMSC (Albumin: green; DAPI: blue; Scale bar = 50 μM). (**E**) Phalloidin fluorescence staining of the arHMSC-Hs demonstrated cortical actin distribution reminiscent of differentiated hepatocytes (Phalloidin: red; DAPI: blue; Scale bar = 50 μM).

To determine the hepatogenic potential of arHMSCs, we induced differentiation of arHMSCs using chemical factors using a modified protocol adapted from Lee *et al*.^20^ (Fig. 4A). arHMSCs started to lose their spindle shape after being exposed to the differentiation media, and gradually adopted a cobblestone morphology on day 14. These cells are named arHMSC-derived hepatocyte-like cells(arHMSC-Hs) (Fig. 4C). To confirm the hepatic differentiation of arHMSCs, we studied the expression of the hepatocyte-specific marker albumin. Immunofluorescence staining results demonstrated that after 14 days of differentiation, arHMSC-Hs expressed albumin (Fig. 4D). The morphological structure of arHMSC-Hs was visualized using actin staining. In contrast to the actin stress fiber observed in arHMSCs (Fig. 3A), cortical actin was dominant in arHMSC-Hs (Fig. 4E), which is reminiscent of primary hepatocytes *in vivo*^18^.

### hepatocyte-specific gene expression

**Figure 5:**
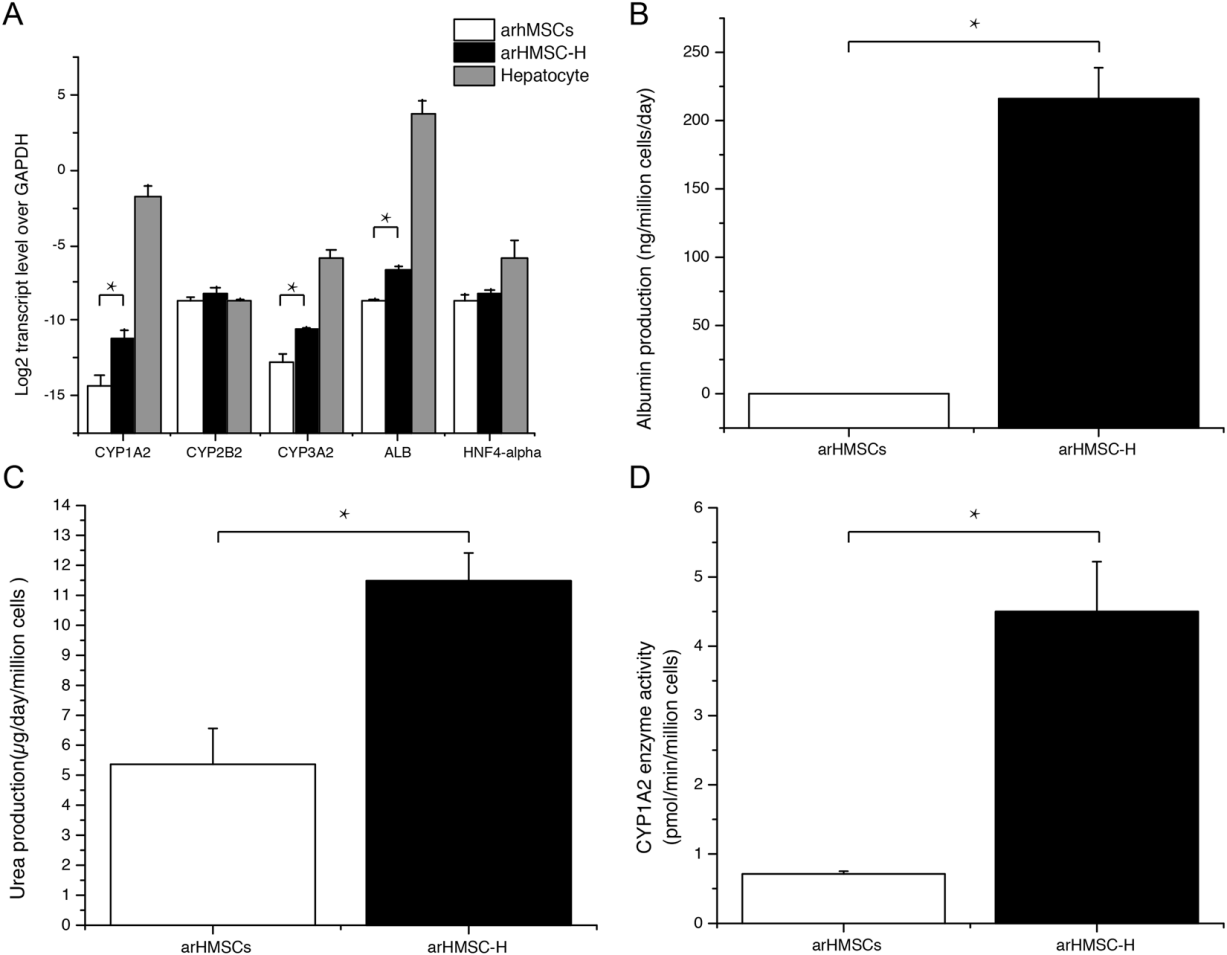
Characterization of arHMSC-Hs. Functional characterization was performed on day 14 of arHMSC differentiation and on day 2 of primary hepatocyte culture in monolayer. (**A**) Analysis of liver specific gene expression. *In vitro* differentiation of arHMSCs induced a significant increase in the transcript levels of CYP1A2, CYP3A2 and albumin. There was no significant difference in the expression of CYP2B1/2 and HNF 4α between arHMSCs, arHMSC-Hs and primary rat hepatocytes. (**B**–**D**) Synthetic and metabolic function of arHMSC-Hs. Urea synthesis, albumin production and enzyme CYP1A2 activity were significantly enhanced after *in vitro* differentiation of arHMSCs. Data plotted as mean ± s.e.m of three independent experiments performed in triplicates An asterisk indicates statistical significance with a confidence interval of 95% (p < 0.05) using Student’s t-test.

To investigate the transition of arHMSCs to hepatic phenotype during the differentiation, we measured the expression of hepatocyte-specific genes CYP (1A2, CYP2B1/2, CYP3A2) for drug metabolism, albumin as a marker of synthetic function and HNF4α for liver-enriched transcriptional factors in arHMSCs, arHMSC-H and mature primary hepatocytes. *In vitro* differentiation of arHMSCs induced a significant increase in CYP1A2, CYP3A2 and albumin expression levels. arHMSC-Hs had 8.7, 4.5 and 200 fold higher expression of CYP1A2, CYP3A2 and albumin, respectively (p = 0.0175, 0.0272, 0.0059 respectively) (Fig. 5B). However, there was no significant difference in the expression of CYP2B1/2 and HNF4α among the three groups. These results indicate that arHMSCs have hepatogenic potential and can differentiate to hepatocytes-like cells.

### Synthetic and metabolic function of arHMSC-Hs

To investigate the functions of arHMSC-Hs, we analyzed albumin production and urea synthesis and compared to arHMSCs (Fig. 5B). *In vitro* differentiation enhanced the urea production from 5.37 ± 1.19 μg/day/million cells for arHMSCs to 11.48 ± 0.93 μg/day/million cells for arHMSC-Hs (p = 0.0249). We also demonstrated that arHMSC-Hs had the ability to produce albumin at a level of 215.96 ± 22.73 ng/million cells/day while albumin production of arHMSCs were undetectable (p = 0.0091) (Fig. 5C). Activity of drug-metabolizing enzyme CYP1A2, one of the most important cytochromes P450 enzymes in rats, was also examined. The arHMSCs expressed lower CYP1A2 activity (0.71 ± 0.04 pmol/min/million cells. arHMSC-Hs expressed a significant enhancement of CYP1A2 activity to 4.35 ± 0.61 pmol/min/million cells (p = 0.0338) (Fig. 5D), which is consistent with the up-regulated CYP1A2 mRNA expression of arHMSC-Hs (Fig. 5A).

### Dose response to paradigm hepatotoxicants

**Figure 6:**
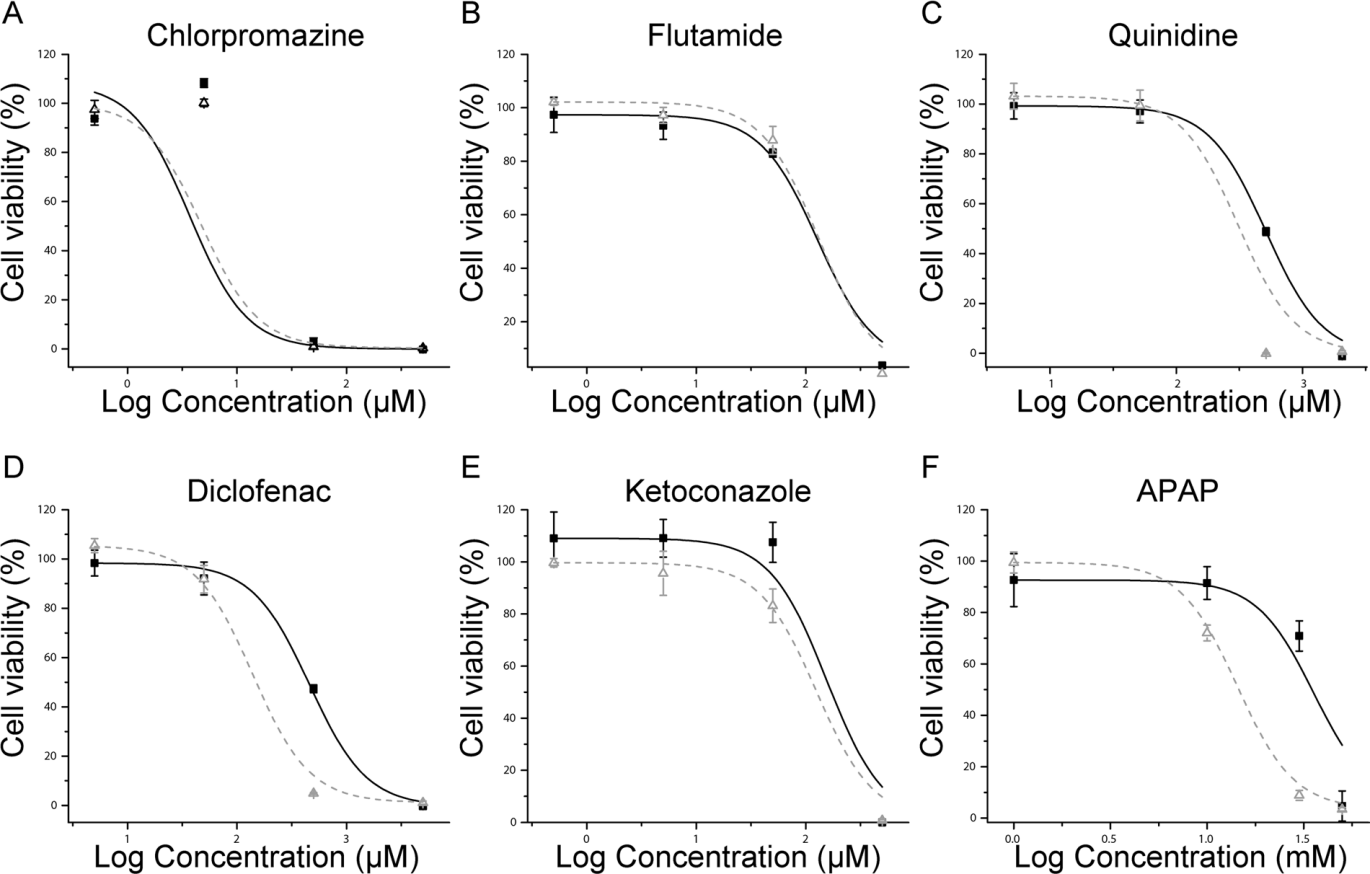
Dose response characteristics of arHMSC-Hs compared with primary rat hepatocytes upon treatment with paradigm hepatotoxicants. (**A**) Chlorpromazine, (**B**) Flutamide, (**C**) Quinidine, (**D**) Diclofenac, (**E**) Ketoconazole and (**F**) APAP. arHMSC-Hs and primary rat hepatocytes exhibited comparable dose response characteristics for chlorpromazine, ketoconazole and flutamide compared to primary rat hepatocytes suggesting the potential use of these cells for studying drug DMPK and toxicity properties. (☐: arHMSC-H, Δ: primary rat hepatocytes). Data plotted as mean ± s.e.m of three independent experiments performed in triplicates from three independent differentiations.

We investigated the dose response characteristics and IC_50_ values of 6 paradigm hepatotoxic compounds with different mechanisms of action, namely acetaminophen (APAP), ketoconazole, diclofenac, chlorpromazine, flutamide and quinidine in the arHMSC-Hs and compared to mature hepatocytes in parallel to study the utility for hepatotoxicity testing (Table 1). arHMSC-Hs and mature primary hepatocytes exhibited similar dose-response characteristics with similar sensitivity for chlorpromazine, ketoconazole and flutamide compared to mature primary hepatocytes, whereas hepatotoxic responses to quinidine, diclofenac and APAP were not as sensitive as mature primary hepatocytes (Fig. 6). This indicates that arHMSC-Hs had similar dose-response characteristics to mature primary hepatocytes for the selected paradigm hepatotoxicants.

**Table 1:** IC50 values measured for 6 paradigm hepatotoxic drugs using arHMSC-Hs and primary hepatocytes.

Drugs	rALMSC-H IC_50_ (µM)	Hepatocyte (µM)	Cmax in rats (µM)	rALMSC-H IC_50_/Cmax
Chlorpromazine	3.6	4.7	0.3^40^	12
Ketoconazole	155.5	125.5	37^33^	4.2
Flutamide	128.4	130.8	24.2^34^	5.3
Quinidine	496.3	298.6	Not available	
Diclofenac	459.9	138.8	33.7^35^	13.6
APAP	35600	14300	728^41^	48.9

## Discussion

We have derived a hepatocyte-like cell source from healthy adult rat livers and characterized its utility for hepatogenic differentiation and drug safety testing *in vitro*. The arHMSCs are derived from the mature hepatocytes without the use of toxic chemicals or reprogramming using retroviruses. Furthermore, the ability to acquire metabolic hepatocyte functions with the ability to expand arHMSC may allow these cells to be exploited as an alternative model for pharmaceutical drug-testing applications or eventually in individualized therapeutic applications.

It is understood that most adult tissues harbor stem cells and progenitor cells, which are capable of differentiating into mature cells of that particular tissue^21^. The best-characterized progenitor cells in liver are oval cells^22,23^. Recent studies have shown that in the adult liver *in vivo*, the mature hepatocytes can dedifferentiate into oval cells, which in turn provides hepatocytes and cholangiocytes^9–12^. These studies provide new hope for deriving hepatocytes from mature hepatocytes by first dedifferentiating hepatocytes into progenitor-like cells and then expand this dedifferentiated progenitor-like cells to act as a source for deriving hepatocyte-like cells. Such an approach could provide an alternate source of hepatocytes for drug screening application.

Here we have also performed a series of experiments to track the derivation of arHMSCs from a mature rat hepatocyte population in an *in vitro* culture. Time-lapse imaging of the hepatocytes, lineage tracking using GFP-Ftractin, demonstrated that arHMSCs originated from mature rat hepatocytes. The time-lapse images demonstrate two distinct phases involved in the dedifferentiation of the rat hepatocytes to arHMSCs. The first phase of dedifferentiation from day 5 of dedifferentiation to day 6 involved cell condensation or shrinkage of the hepatocyte islands after which arHMSCs appeared from these clustered cells and proliferated to give rise to a homogenous arHMSC culture. Interestingly the cells expressed mesenchymal markers such as vimentin, alpha-smooth muscle actin and were positive for CD90, CD44 and CD29. The cells were however negative for CD45 (Fig. 2B), a hematopoietic marker^24^. Our arHMSCs were different from that derived by other groups, and predominantly expressing mesenchymal markers. The role of EMT in liver development and regeneration is well understood^25^. Here we confirmed the role of EMT in the derivation of arHMSCs from mature rat hepatocytes.

Having demonstrated and characterized the origin and the phenotype of arHMSCs from the adult rat hepatocytes, we further demonstrate the ability of arHMSCs to differentiate into hepatocyte-like cells and application for studying dose-response characteristics. arHMSC-Hs expressed lower hepatocyte-specific functions compared to those observed in mature hepatocytes. This is similar to findings from other groups that have attempted to differentiate embryonic stem cells (ESC) or induced pluripotent cells (iPS) cells into mature hepatocytes and observed fetal hepatocyte-like functionality^26–29^. This highlights a limitation of directed differentiation protocols in producing mature hepatocytes. Previous differentiation studies of arHMSCs to hepatocytes have shown contradictory results. Chen *et al*. could not derive hepatocytes from their progenitors, whereas the work by Sahin *et al*.^14^ could show directed differentiation of the liver derived progenitor cells (LDPCs) to hepatocytes; however, they were not characterized for their metabolic function and drug-testing applications. The work by Maerckx *et al*., had demonstrated that the differentiated rat progenitor cells express albumin but not important hepatic specific markers such as HNF^15^. During differentiation, our arHMSCs undergo morphological changes from a mesenchymal spindle-shaped morphology to a more epithelial morphology similar to hepatocytes^30^. arHMSC-Hs were positive for albumin, an important protein synthesized by mature hepatocytes. arHMSC-Hs also demonstrated increased urea secretion, an important hepatocyte-specific function that is not detected in arHMSCs and other progenitor cells. The levels of albumin secretion in our differentiated arHMSC-Hs were lower than that described by Sahin *et al*. This could be due to the characteristics of the progenitor cells expressing CD 45 in their study that were rounded and morphologically distinct from the arHMSCs described in our work^14^. Further, their albumin secretion was estimated in 2-weeks conditioned media while we tested albumin secretion after a 24 hour conditioning. The differentiated arHMSCs demonstrated significantly increased expression levels of CYP 1A2, 3A2 and albumin compared to the undifferentiated arHMSCs. arHMSC-Hs retained similar HNF4 alpha expression levels as arHMSCs, which is an early hepatocyte marker. These differentiated markers were significantly lower compared to the mature primary hepatocytes. However, this is similar to what has been observed with current differentiation protocols using ES and iPS cells wherein the hepatocytes obtained express fetal hepatocyte-like markers and are not mature when compared to primary human hepatocytes^28^. The expression of hepatocyte-specific gene and phenotypic markers in *in vitro* differentiated arHMSCs offers potential to study dose-response characteristics of selected paradigm hepatotoxicants for drug screening applications. The differentiated arHMSCs demonstrated similar dose-response characteristics compared to adult rat hepatocytes for the 3 drugs chlorpromazine, ketoconazole and flutamide. The arHMSC-Hs demonstrated a 2 fold lower sensitivity for quinidine, diclofenac and APAP. Different cell sources (either from different organism or derived from different source such as iPSC or ESC) have different sensitivity to different drugs^29,31^. In the current study, since the derived hepatocyte-like cells are similar but not entirely the same as the mature hepatocytes in functionality, there are some differences in sensitivity for different hepatotoxicants. The IC_50_ obtained for 5 of the 6 hepatotoxicants tested was within 50*Cmax which is comparable to that obtained with mature primary hepatocytes^32–36^ which is the current gold standard for hepatotoxicity testing *in vitro* making this cell type a suitable model for evaluating concentration responses^37^.

In conclusion, our study demonstrates the origin of arHMSCs from the adult hepatocytes and the utility of these self-renewing cells to derive hepatocyte-like cells for hepatotoxicity screening. Further efforts are needed to improve the maturity of these differentiated arHMSCs to enhance its hepatocyte-specific functions for use in therapeutic applications.

## Materials and Methods

All chemicals were purchased from Sigma Aldrich Singapore unless otherwise stated.

### Collagen coating

Neutralized collagen solutions of 1.5 mg/mL & 0.5 mg/mL were prepared by mixing Type I bovine dermal collagen (INAMED BioMaterials Corp, USA), 0.1 M sodium hydroxide (NaOH), 10× phosphate buffered saline (PBS) and 1× PBS. Plates for arHMSCs differentiation and primary hepatocyte culture were coated with 1.5 mg/mL and 0.5 mg/mL neutralized collagen respectively at room temperature for at least half an hour and incubated at 37 °C for an hour.

### Rat hepatocyte isolation

Rat primary hepatocytes were isolated from male Wistar rats weighing between 200 g to 300 g using a two-step *in situ* collagenase perfusion method^38^. 200–300 million cells (viability > 90%, Trypan Blue exclusion assay) were harvested from each rat. The cells obtained were subjected to fractionation to purify the hepatocytes from the non-parenchymal cells. However, we did not perform flow cytometry to isolate and plate the pure hepatocyte population. The animals were obtained from InVivos, Singapore. All experiments were carried out in accordance to the IACUC protocol approved by the IACUC committee of the National University of Singapore; protocol number R15–0027.

### arHMSCs isolation and culture

Primary hepatocytes were seeded at a density of 4 × 10^4^ cells/cm^2^ onto 0.5 mg/mL collagen coated flasks in Williams’ E media supplemented with 1 mg/mL bovine serum albumin (BSA), 0.5 mg/mL insulin, 5 nM dexamethasone, 50 ng/mL linoleic acid, 100 units/mL penicillin and 100 mg/mL streptomycin (P/S) at 37 °C in a humidified atmosphere containing 5% CO_2_. Culture medium was changed after 4 hours to Dulbecco’s modified eagle’s medium (DMEM) low glucose (Gibco, Singapore) supplemented with 3500 mg/L D-glucose, 1200 mg/L HEPES, 1300 mg/L sodium bicarbonate, 10% FBS and 1% P/S. Media was refreshed every 3–4 days thereafter. arHMSCs began to appear and proliferate after 5 days in culture. The cells were then trypsinized 7 days after culture with 0.25% trypsin-EDTA after reaching 90% confluence, and re-plated at 1 × 10^4^ cells/cm^2^ on collagen-coated flasks.

### Hepatogenic differentiation of arHMSCs

arHMSCs (from the fourth passage onwards) were seeded on collagen coated plates at a density of 1.4 × 10^4^ cells/cm^2^. Cells were maintained in DMEM supplemented with 10% FBS and 1% P/S until they were 80–90% confluent. For hepatogenic differentiation, DMEM media was switched to basal differentiation medium containing 60% DMEM low glucose (Gibco, Singapore), 40% MCDB 201 water, 0.25× linoleic acid – bovine serum albumin, 0.5% fetal bovine serum (FBS), 0.25× insulin-transferrin-selenium, 100 IU/mL penicillin, 100 mg/mL streptomycin (Cellgro 30–002-CI, USA), 0.1 mM, L- ascorbic acid, 10^−3^ μM dexamethasone and 55 mM 2-mercaptoethanol (Gibco, Singapore)^39^. Undifferentiated arHMSCs were incubated with basal differentiation media supplemented with 20 ng/mL epidermal growth factor (EGF) (R&D Systems, USA) and 10 ng/mL fibroblast growth factor-4 (FGF-4) (R&D Systems, USA) for pre-differentiation for 2 days. Thereafter, cells were induced to differentiate for 7 days with basal differentiation media containing 20 ng/mL hepatocyte growth factor (HGF) (R&D Systems, USA), 10 ng/mL FGF-4 and 0.61 g/L nicotinamide. For subsequent maturation steps, cells were treated with basal differentiation media supplemented with 20 ng/mL hepatocyte growth factor (HGF) (R&D Systems, USA), 20 ng/mL oncostatin M (R&D Systems, USA), 0.61 g/L nicotinamide and 1 μM dexamethasone for 7 days. Culture medium was changed every 2 days.

### Live imaging

Cells were seeded in collagen coated plates and continuously tracked using live cell imaging and analysis platform Cell-IQ^®^ (CM Technologies Oy, Finland), which maintained the cells at 37 °C with 5% CO_2_ and monitored the cells using phase contrast microscopy. Cells were imaged continuously for 7 days.

### Transfection and time lapse imaging of hepatocytes

Primary hepatocytes were seeded at a density of 4 × 10^4^ cells/cm^2^ onto 0.5 mg/mL collagen coated 6 well plate in supplemented Williams’ E media (as described above) and cultured at 37 °C in a humidified atmosphere containing 5% CO_2_ for 2 hours to allow hepatocytes to attach. Hepatocytes were than transfected with GFP/GFP-Ftractin plasmid using Lipofectamine® 2000 according to the manufacturer’s protocol. Briefly plasmid (2 µg) was mixed with 4 µL lipofectamine in 500 µL supplemented WE media (devoid of antibiotics). This solution was incubated at room temperature for 20 minutes and added to hepatocytes cultured in 6 well plate and incubated for 3 hr. Hepatocytes were then washed in 1 × PBS and culture medium was changed to DMEM low glucose (Gibco, Singapore) supplemented with 3500 mg/L D-glucose, 1200 mg/L HEPES, 1300 mg/L sodium bicarbonate, 10% FBS and 1% P/S. Media was refreshed every 3–4 days thereafter. The cells were imaged using the EZ Olympus microscope from day 4 to day 7 at an interval of 4 hours. Subsequently, the cells were stained (as described in section 2.8) and were imaged using the EZ Olympus microscope. The same cells were imaged during live cell tracking and after fixation.

### Flow cytometry

arHMSCs were trypsinized and suspended at a concentration of 1.5 × 10^4^ cells/μL in 1× PBS with 10% fetal bovine serum (FBS) (Biowest, USA). 95 μL cell suspension and 5 μL antibody (CD29- PE, CD44-FITC, CD45-FITC CD90-FITC, ASGPR- PE) (BD Pharmingen, Singapore) were mixed and incubated at 4 °C for 30 min. Cells were washed three times and analyzed with a FACSCanto II flow cytometer (BD LSRFortessa^TM^, USA). The data was plotted and analyzed using FlowJo (Tree Star Inc., USA).

### Fluorescence staining

arHMSCs and arHMSC-Hs were fixed with 3.7% paraformaldehyde (PFA) for 20 min at 37 °C and permeabilized thereafter with 0.1% Triton X-100 in PBS for 10 min. Nonspecific binding was prevented by blocking the permeabilized cells for 1 hour in a PBS solution containing 2% BSA at room temperature. Cells were then incubated with primary antibodies (anti-alpha smooth muscle actin antibody (α-SMA), phalloidin (Molecular Probes, USA), anti-vimentin, anti-albumin (Abcam, UK), anti-cytokeratin 19 (Abcam, UK), N-Cadherin (Life technologies, USA) in the PBS solution with 2% BSA (1:100) overnight at 4 °C. Cells were then washed three times the next day with gentle shaking in PBS and incubated for 2 hours with either rabbit or mouse secondary antibodies (Bioworld, USA) at room temperature. After washing three times in PBS, nuclei were stained for 5 min with the nuclear dye DAPI (1:1,000). After three rinses, samples were imaged using confocal microscopy (Fluoview FV 500, Olympus, USA) using a 10× air & 60× water lens. Images were processed using IMARIS (Bitplane Technologies, USA).

### Hepatocyte functional assays

Albumin production was measured using a rat albumin ELISA quantitation kit (Bethyl laboratories Inc., USA). Urea synthesis was measured using a urea nitrogen kit (Stanbio laboratory, USA). For CYP1A2 activity analysis, CYP specific probe substrate (200 μM phenacetin) was diluted in Krebs-Henseleit buffer (KHB) and incubated with the cells for 1.5 hours. The supernatant were collected and stored at −80 °C until being measured by liquid chromatography-mass spectrometry (LC/MS). All functional data were normalized to one million cells by using a Quant-iT^TM^ picoGreen dsDNA assay kit (Invitrogen, Singapore).

### Quantitative reverse-transcription polymerase chain reaction (qRT-PCR)

Total RNA was isolated using a Total RNA kit I (Omega, USA) and was converted to cDNA using an iScript^TM^ cDNA synthesis kit (Biorad, USA). 12.5 ng of cDNA was used to perform qRT-PCR using SYBR green I Master (Roche, USA). Gene expression was performed using rat specific primers and normalized to GAPDH. Data was plotted as a Log_2_ transcript over GAPDH.

### Drug-induced hepatotoxicity

Primary rat hepatocyte cultures were used for drug-testing one (1) day post seeding and differentiated arHMSCs were used for hepatotoxicity testing after 14 days of differentiation and maturation. Cells were treated with APAP, diclofenac, ketoconazole, chlorpromazine, flutamide or quinidine at four different concentrations for 24 hours. The drugs were dissolved in DMSO and the final concentrations of DMSO in the medium were kept at less than 0.1%. Blank and drug sample have equal amount of DMSO (equivalent to DMSO at the highest drug concentration). Cell viability was measured by MTS assay using the CellTiter 96 Aqueous One solution (Promega, USA) which is routinely a used method to measure hepatocyte toxicity.

### LC/MS measurement of CYP specific metabolites

300 μL of sample containing metabolites were mixed with 100 μL of 100 ng/mL internal standard APAP-D4 and dried using a concentrator at 30 °C (Eppendorf, Germany) under vacuum. The dried residues were reconstituted using 100 μL methanol containing 0.1% formic acid and centrifuged at 10, 000 rpm for 10 min at 4 °C. 80 μL of supernatant was then transferred to the LC/MS sample vials and used for measurement by LC/MS system (LC: 1100 series, Agilent, Singapore; MS: LCQ Deca XP Max, Finnigan, Singapore) with 100 × 3.0 mm onyx-monolithic C18 column (Phenomenex, USA). The mobile phase consisted of solvent A (0.1% formic acid in water) and solvent B (0.1% formic acid in methanol) with a flow rate of 0.8 mL/min. The elution scheme for the measurement of acetaminophen involved solvent B that was gradually increased from 6% to 90% over 6 min. The MS parameter settings were as follows: spray voltage 5 kV; sheath gas flow rate: 80; auxiliary gas flow rate: 20; capillary temperature: 350 °C; tube lens: 45 V; and capillary voltage: 30 V.

### Statistical method

The Student’s *t*-test was used to analyze the statistical significance of the data between two groups. Sign test was used to analyse the difference in morphology of individual cells during the course of EMT. For drug testing, we generated three independent arHMSC lines (from different lot of rat hepatocyte isolated on different days) and the data presented represents a mean on such three independent arHMSC lines. Values with *p* < 0.05 were considered statistically significant.

## Electronic supplementary material

Supporting Figures
