# Supplementary material for: Directed Differentiation of Adult Liver Derived Mesenchymal Like Stem Cells into Functional Hepatocytes
Source: Sci Rep. 2018 Feb 12;8:2818. doi: 10.1038/s41598-018-20304-5 (PMC5809507; doi:10.1038/s41598-018-20304-5)
Supplement: Supplementary file 1 — Supporting Figures [file 41598_2018_20304_MOESM1_ESM.pdf]

Directed Differentiation of Adult Liver Derived Mesenchymal Like Stem Cells into Functional Hepatocytes

Xiaobei Luo[1], Kapish Gupta[2], Abhishek Ananthanarayanan[3,4,5],Zenan Wang[1,6], Xia Lei[7], Aimin Li[1], Rashidah Binte Sakban[7], Side Liu[1 *] , Hanry Yu [1,2,4,5,7,8 *]

[1]Department of Gastroenterology, Nanfang hospital, Southern Medical University, Guangzhou, China.

[2]Mechanobiology Institute, National University of Singapore, Singapore.

[3]Invitrocue Pte Ltd, Singapore.

[4]Institute of Bioengineering and Nanotechnology, Agency for Science, Technology and Research (A*STAR), Singapore

[5]NUS Graduate School for Integrative Sciences and Engineering, Centre for Life Sciences (CeLS), Singapore, Singapore

[6]Department of Gastroenterology, Beijing Chao-Yang Hospital, Capital Medical University, Beijing, China.

[7]Department of Physiology, Yong Loo Lin School of Medicine, National University of Singapore, Singapore, Singapore.

[8]BioSyM, Singapore-MIT Alliance for Research and Technology, Singapore

*Corresponding author:

1. Side Liu (liuside@163.com)

Department of Gastroenterology, Nanfang Hospital, Southern Medical University, Guangzhou 510515, China.

2. Hanry Yu (hanry_yu@nuhs.edu.sg)

MD 9 #04-11, 2 Medical Drive, National University of Singapore, Singapore 117593 Tel.

No. + 65 65163466, Fax No. +65 68748261

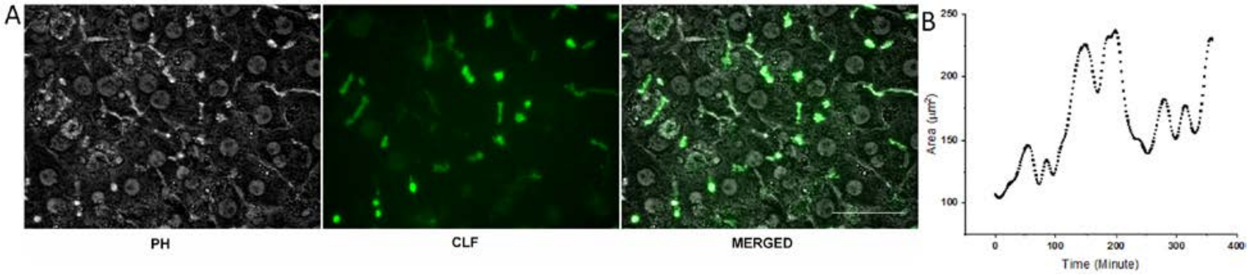

Supporting Figure1: Bile canaliculi were used as a marker for polarized mature hepatocytes in isolated cells. Bile canaliculi can be seen as bright structure in phase contrast images as shown in Figure A. Cells were incubated in CLF (Cholyl-Lys-Fluorescein, a fluorescein-labeled bile acid analog) and within 10 minutes CLF can be seen localizing in the BC (co-localized with bright structure of Phase contrast). These bright structures are very dynamic and can be observed to rapidly expand and contract as shown by area change curve in B.

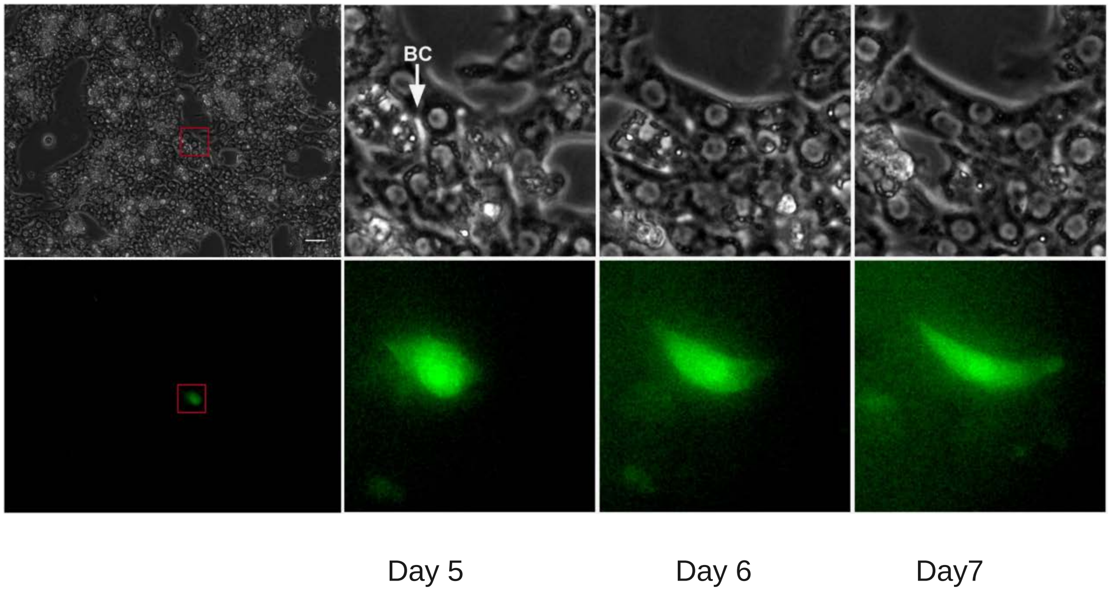

Supporting Figure 2: Hepatocytes show cobblestone morphology and association with bile canaliculi (BC) (day 5). By day 7 the cell show change in morphology to fibroblast (elongated cell) type with disappearance of BC showing loss of hepatic morphology and function. A Similar example is shown in the Figure 2 in the manuscript. It also shows that that with appearance of fibroblast like morphology the cell also start to express mesenchymal and progenitor like marker.
